# Examining nursing students’ awareness of various medical errors during clinical internships to enhance patient safety: A multi-center cross-sectional study

**DOI:** 10.1371/journal.pone.0311681

**Published:** 2024-12-31

**Authors:** Ola Mousa, Basma Salameh, Asmaa Saber Ghaly, Md Gulzarull Hasan, Aishah Abdulrahman Almefarfesh, Sheeba Kumari, Mashael Huwaikem

**Affiliations:** 1 Faculty of Nursing, Minia University, Minia, Egypt; 2 Faculty of Nursing, Arab American University, Jenin, Palestine; 3 College of Applied Medical Sciences, King Faisal University, Al-Hasa, Saudi Arabia; 4 Department of Data Science, Prasanna School of Public Health, Manipal Academy of Higher Education, Manipal, India; 5 Cinical Nutrition Department, College of Applied Medical Sciences, King Faisal University, Al Ahsa, Saudi Arabia; University of Lisbon: Universidade de Lisboa, PORTUGAL

## Abstract

**Background:**

Patient safety is a global concern within health facilities, primarily attributed to medical errors, constituting a significant global public health issue. Patients experiencing medication errors face serious problems, with increasing mortality rates and escalating hospital costs.

**Aim:**

The study aims to examine nursing students’ awareness of various medical errors during their clinical internships at Al- Ahsa, identifying types of errors to enhance patient safety.

**Methods:**

A cross-sectional study was conducted among 160 nursing students during their internship at King Faisal University, Al Ahasa, Saudi Arabia.A total of 131 participants completed a self-administered questionnaire based on a medical error scale consisting of 43 items across six subscales. Data were analyzed using SPSS version 22. The outcomes of interest included falls, blood and blood Products transfusion, medication practices, care practices, communication, and controlled practices.

**Results:**

Overall, nursing students scored 188 out of 215. Among the sub-dimension scores, medication practices received the highest mean score, while Patient falls emerged with the lowest score. In our study, students ranked falling as the least significant factor.

**Conclusion:**

Study findings indicate that nursing students exhibit a low tendency to commit medical errors, which is encouraging for the future implementation of patient safety protocols. This trend suggests that nursing students are developing strong competencies in safe nursing practices, which contributes to reducing the incidence of medical errors and improving overall patient safety in clinical settings.

## Introduction

In low- and middle-income countries (LMICs), unsafe healthcare practices cause an estimated 134 million adverse health events yearly, resulting in approximately 2.6 million fatalities. Additionally, one out of 10 admitted patients falls victim to medical errors (MEs) associated with healthcare delivery [1].

Patient safety is a serious global public health concern [2]. According to the World Health Organization (WHO), it is estimated that 1 in 10 patients is harmed while receiving hospital care in high-income countries, with 1 in 300 patients succumbing to this harm. Using conservative estimates, recent data reveals that patient harm ranks as the 14th leading cause of morbidity and mortality worldwide [1]. Contemporary healthcare systems highlight the paramount of preserving patient safety, a factor influenced by various elements. Among the most north worthy factors affecting patient safety are medication errors (MEs) by healthcare providers [3, 4]. Furthermore, it was shown that 50% of these incidents were deemed preventable. 83% of hospital admissions in low- and middle-income countries were considered preventable, with 8% of these cases involving patient harm [5].

Regardless of its visibility or harmful the error is to the patient; a medical error is a preventable consequence resulting from medical care. “Adverse medication events, incorrect transfusions, misdiagnosis, under- and overtreatment, surgical injuries, wrong-site surgery, suicides, injuries or deaths from restraints, falls, burns, pressure ulcers, and instances of mistaken patient identities” are common problems during healthcare provision. Emergency departments, intensive care units, and operating rooms are the settings where incidents with high mistake rates and severe consequences are most likely to occur. Extremes of age, the introduction of new procedures, urgency, and the severity of the medical condition being treated are also associated with medical errors [6, 7].

Medical errors are unfortunately common and result in extraordinary costs. It is crucial errors are openly discussed and seen as learning opportunities. However, medical schools have been slow to integrate curricula addressing medical errors or providing training in how to respond to them. Due to incomplete error disclosure, students may lack both formal and informal education in error management [8]. Efforts to increase medical student error reporting can potentially be achieved through patient safety education, culture change, and teaching students how to report errors [9].

According to various studies, about one-third of medicine complications are due to MEs [4, 10, 11]. Medication errors pose serious problems for patients, leading to an increase the mortality rates and hospital cost. In Saudi Arabia the most common errors are prescribing errors, followed by administration errors. The total incidence of MEs in Saudi Arabia hospitals was estimated at 44.4% [12]. Adverse Drug Events (ADEs) were common in Saudi hospitals, especially in ICUs, causing significant morbidity and mortality [13].

In order to change the culture of healthcare organizations to one focused on patient safety, medical students should be equipped to recognize unsafe conditions, systematically report errors and near misses, investigate and improve such systems with a thorough understanding of human fallibility, and disclose errors to patients [14]. Early in their medical education, they should be taught about human error and the factors influencing adverse events [14]. Although MEs may occur across various health care professions, their incidence is notably high in nursing, a practice-oriented profession, compared to other medical professions [15, 16]. In the nursing profession, nursing students are exposed to more risks and clinical errors during their clinical activities due to their underdeveloped skills, limited clinical experience and lack of knowledge [17].

Examining nurses’ reactions to MEs in healthcare practice revealed that majority of them regarded these errors as insignificant to patient safety. The researchers observed that this attitude, considering mistakes as minor or negligible, tend to cause many complications in healthcare settings [18]. Unintended MEs have a significant impact on the victims, contributing to both mental and adverse emotional effects in healthcare providers [19].

Understanding patient safety can be advanced by examining certain related topics, including the significance of reporting errors, the systems approach to ensuring patient well-being, and the investigation of error causation in medicine. It is crucial to encourage transparency within the organization and extend it beyond to gain insights from adverse events. In this context, the disclosure of errors (or potential errors) is crucial, and all healthcare workers should be involved in this process [20].

Developing a culture of safety is a fundamental element in many initiatives aimed at enhancing the quality of patient care [21, 22]. Previous research studies have demonstrated a correlation between the safety culture and the behaviors of clinicians in terms of greater reporting of errors, decreased adverse events, and decreased mortality. Key components of fostering a strong safety culture include encouraging the open discussion of safety issues without fear of blame or retribution, implementing feedback mechanisms to communicate improvements made after safety concerns, and promoting transparency [23]. The nursing curriculum typically covers the theoretical needed for clinical practice but often lack emphasis on the application of safety knowledge and competencies essential for providing safe patient care [24]. To enhance the safety culture, patient safety education must be incorporated into all nursing courses, using diverse teaching strategies such as simulation, online modules, and virtual reality which will enhance student’s competency toward safe medication administration [25–27]. As medical errors and patient safety continue to evolve in both academic and clinical settings, curricula should include targeted training on these critical issues [28].

Therefore, it is essential to educate nursing students about medical error and their consequences. Incorporating these elements across courses will reinforce safety behaviors and foster a proactive attitude toward patient care. In this context, the present study aimed to examine nursing students’ awareness of various medical errors during their clinical internship in Al- Ahsa and to identify the types of medical errors that occur during such internship program. The findings on student awareness will provide valuable insights into the strengths and weaknesses of the nursing curriculum, which can inform the development and implementation of tailored educational programs tailored for nursing interns to address medical errors. These programs could improve interns’ understanding of medical errors, their associated risk, and preventative measures. Additionally, these insights will highlight areas where nursing curriculum may need enhancement.

## Methods

### Study design

A cross-sectional study was conducted between July and November 2021 in hospitals that accept the intern students "King Fahd- MCH-Ben Jalawy- King Faisal- Psychiatric.

### Sampling techniques

Self-administered survey was administered among internship students at College of Applied Medical Sciences, King Faisal University, Al Ahasa, Saudi Arabia. Clinical Nursing students (interns) enrolled during the study period were eligible to participate (n = 160). Using Raosoft software, a sample size of 114 was calculated using a 50% response distribution, a 95% confidence interval, and a 5% margin of error. However, 131 students in all ultimately completed the questionnaires, resulting in a response rate of 81.88%.

Participants were all students who had worked in hospital settings for a minimum of one month and had been involved in patient care. During clinical time, students completed an anonymous, self-administered questionnaire. The study participants signed a consent form and agreed to participate.

### Data collection tool

After reviewing many studies, the researchers adopted the Medical Error Scale for Nurses [29]. It consists of 43 items distributed across six subscales: falls, blood and blood products transfusion, medication practices, care practices, communication, and other controlled practices.

The questionnaire was composed of three sections: The first section includes sociodemographic characteristics of participants including gender, age, and how many months in internship. Second section is three open-ended questions related to reporting of errors including: Did you make any medical errors, did you report any medical errors, and What is the type of error that you made (Mix medication, Needle stick injury, None, Wrong Dose, Wrong insertion of cannula, and Wrong route). Finally, third section is about errors in clinical application. 43 items in six subscales: “12 items in Factor 1‐Falls, six items in Factor 2‐ Blood and Blood Products Transfusion, six items in Factor 3‐Medication Practices, eight items in Factor 4‐Care Practices, five items in Factor 5‐Communication, and six items in Factor 6‐Other Controlled Practices”. A 5-point Likert scale was used for the third part. Responders specify their level of agreement to a statement typically in five points: “Always” (5), “Often” (4), “Sometimes” (3), “Rarely” (2), and “Never” (1). **“**A score close to 215 (maximum score 43 × 5) indicated the nurse was disciplined or cautious about medical errors, whereas a score close to 43 (minimum score 43 × 1) indicated that he/she was not careful about medical errors or was at risk of making medical errors”. To facilitate comparisons, the mean scores for the overall scale and each of its subscales were divided by the total number of items, resulting in values ranging from 1 to 5 for both the subscales and the overall scale.

The reliability for the questionnaire was determined using Cronbach’s Alpha (0.89). Additionally, the questionnaire was tested for content validity by 5 experts: 3 holding PhDs in the nursing field and 2 clinical instructors working in the ICU. They were asked to assess its accuracy, provide suggestions to enhance clarity, and determine whether the items appropriately represented the primary objectives of the study.

### Data collection methods

Data was gathered at the culmination of the fall semester, immediately following the completion of their internship. The researchers conducted a comprehensive briefing with participants, emphasizing the importance of their contributions to the study’s objectives. The students were assured of their right to withdraw from the study at any time. Prior to participation, all students provided explicit consent form, affirming their willingness to contribute to the research. The students completed paper-based, English-language questionnaires distributed in person by the researcher. The data was started on 20/07/2021 and ended on 30/11/2021.

### Ethical considerations

The study gets the approval from the ethical research board at King Fahd Hospital "IRB KFHH" RCA No: 20-EP-2021.

Following the explanation of the aim of the study, the internship students gave their written informed consent. Respondent anonymity was guaranteed, and confidentiality was ensured. Additionally, access to secure data is restricted only to the study team.

### Data management and analysis plan

Data collected from the survey were analyzed using JAMOVI software. Descriptive statistics were computed to summarize the characteristics of the study population and their responses to the questionnaire. These statistics included means and standard deviations for continuous variables, such as age and months of internship experience, as well as frequencies and percentages for categorical variables. This approach facilitated the description of response distributions across various categories, including the frequency of medical errors, types of errors, and responses to Likert scale items.

To explore the relationships between variables, specific statistical tests were utilized. The chi-square test of association was employed to determine whether a statistically significant association existed between the level of internship experience and the likelihood of making or reporting medical errors. The results of the chi-square test were evaluated using the p-value, with a significance threshold set at 5% (p < 0.05). Additionally, the Gamma statistic was calculated to assess the strength and direction of the association between levels of experience and the frequency of medical errors.

Moreover, correlation analysis was performed to examine the relationships between the various subscales of the medical error scale. Pearson correlation coefficients were calculated to determine the degree of linear association. These analyses were essential in understanding the factors contributing to medical errors. The results of all statistical tests were meticulously interpreted to identify significant findings related to the frequency and types of medical errors, as well as the factors that influence the likelihood of reporting errors.

## Results

Out of 160, 131 participants completed the questionnaire. The age of participants in years and their working experience in months are presented in Table 1. The mean age of participants who participated in the study is 23.48 years, with a standard deviation of 0.80 years. The maximum and minimum ages are 22 and 27, respectively. The mean experience of participants in the hospital is 3.15 months; the minimum and maximum are 1 and 12 months, respectively.

**Table 1:** Demographic data of the participants.

	Age in complete years (Count 131)	Months of working in hospital (Count 131)
**Mean**	23.48	3.15
**std**	0.80	2.54
**min**	22	1
**max**	27	12

Table 2 shows the number and percentage of responses to the questionnaire questions. Respondents were asked 43 questions in total.

**Table 2:** Errors in clinical application.

	Questions number	Questions	Frequency					Percent				
			Always	Never	Often	Rarely	Sometimes	Always	Never	Often	Rarely	Sometimes
Factor 1- Falls	Q1.	I put the patient bell in a place where the patient can easily reach	91	2	20	5	13	69.47	1.53	15.27	3.82	9.92
	Q2.	I always inform the patient to call the nurse when she wants to stand up.	70	1	39	1	20	53.44	0.76	29.77	0.76	15.27
	Q3.	I put pillows on the bed edges of agitated patients	71	3	28	4	25	54.20	2.29	21.37	3.05	19.08
	Q4.	I take unused materials from the patient room.	48	19	28	10	26	36.64	14.50	21.37	7.63	19.85
	Q5.	I always lock beds or wheelchairs when not using	73	6	34	4	14	55.73	4.58	25.95	3.05	10.69
	Q6.	I constantly monitor patients during transport and transfer	86	1	24	0	20	65.65	0.76	18.32	0.00	15.27
	Q7.	Patient companions tell the nurse when they leave	49	2	40	6	34	37.40	1.53	30.53	4.58	25.95
	Q8.	If the patient gets up for the first time, I will surely accompany him	83	4	23	5	16	63.36	3.05	17.56	3.82	12.21
	Q9.	I visit a patient at risk of falling frequently	73	4	30	4	20	55.73	3.05	22.90	3.05	15.27
	Q10.	I monitor the place if the floor is wet in patient rooms / corridors	73	5	32	2	19	55.73	3.82	24.43	1.53	14.50
	Q11.	I monitor the patient after anti-hypertensive and sedative medications	74	6	19	6	26	56.49	4.58	14.50	4.58	19.85
	Q12.	I inform patients and their companions about the causes and precautions of falls	75	3	28	2	23	57.25	2.29	21.37	1.53	17.56
Factor 2‐ Blood and Blood Products Transfusion	Q13.	I apply blood and its products according to the suitable technique	88	1	25	0	17	67.18	0.76	19.08	0.00	12.98
	Q14.	I verify the patient’s blood type information before applying blood and blood products	87	1	24	1	18	66.41	0.76	18.32	0.76	13.74
	Q15.	I check the label information before applying blood and blood products	92	4	20	3	12	70.23	3.05	15.27	2.29	9.16
	Q16.	Before I apply blood and blood products I identify the patient	88	7	18	3	15	67.18	5.34	13.74	2.29	11.45
	Q17.	I prepare blood and blood products according to the procedure	86	8	19	1	17	65.65	6.11	14.50	0.76	12.98
	Q18.	After applying blood and blood products, I monitor the patient for complications	85	4	23	2	17	64.89	3.05	17.56	1.53	12.98
Factor 3‐Medication Practices	Q19.	I administer the drug after I have verified the patient’s id	90	2	20	4	15	68.70	1.53	15.27	3.05	11.45
	Q20.	I know the injection sites and make the drug to the right area	88	1	22	3	17	67.18	0.76	16.79	2.29	12.98
	Q21.	I do not prepare drugs without checking the physician order	82	4	18	1	26	62.60	3.05	13.74	0.76	19.85
	Q22.	I record my drug applications	90	1	19	4	17	68.70	0.76	14.50	3.05	12.98
	Q23.	I prepare drugs according to aseptic technique in oral drug application	90	2	22	3	14	68.70	1.53	16.79	2.29	10.69
	Q24.	I prepare sterile drugs for parenteral drug administration	86	1	25	2	17	65.65	0.76	19.08	1.53	12.98
Factor 4‐Care Practices	Q25.	I monitor the local impact of my care practices	73	1	34	0	23	55.73	0.76	25.95	0.00	17.56
	Q26.	I monitor the systemic effects of my care practices	71	1	39	2	18	54.20	0.76	29.77	1.53	13.74
	Q27.	I check and verify the patient’s identity information in my applications.	85	1	32	3	10	64.89	0.76	24.43	2.29	7.63
	Q28.	Immediately notify the physician of any abnormal conditions that I have observed	85	1	33	3	9	64.89	0.76	25.19	2.29	6.87
	Q29.	Before application, I will check if the tool / equipment I will use is working correctly	92	3	24	2	10	70.23	2.29	18.32	1.53	7.63
	Q30.	Knows the effect of care practices on the patient	86	3	28	0	14	65.65	2.29	21.37	0.00	10.69
	Q31.	I follow the patient regularly	72	4	34	4	17	54.96	3.05	25.95	3.05	12.98
	Q32.	I perform patient monitoring "follow up" as needed	85	1	27	5	13	64.89	0.76	20.61	3.82	9.92
Factor 5‐Communication	Q33.	I share information about patient care and results during shift changes	84	4	23	4	16	64.12	3.05	17.56	3.05	12.21
	Q34.	I verify non-explicit requests / orders that may cause problems	77	4	29	0	21	58.78	3.05	22.14	0.00	16.03
	Q35.	I record all information about the patient’s treatment to nurse observation	79	1	36	2	13	60.31	0.76	27.48	1.53	9.92
	Q36.	Communicate patient’s complaints / problems to healthcare team members immediately	83	1	34	1	12	63.36	0.76	25.95	0.76	9.16
	Q37.	I dispose of the materials according to the color of the medical waste bins	74	1	36	2	18	56.49	0.76	27.48	1.53	13.74
Factor 6‐Other Controlled Practices	Q38.	I do not leave the medication with the patient to drink / apply the medication.	84	4	23	2	18	64.12	3.05	17.56	1.53	13.74
	Q39.	I don’t apply the medication prepared by someone else to the patient.	77	3	20	9	22	58.78	2.29	15.27	6.87	16.79
	Q40.	In case of oral medication, I stay with him until he drinks it.	74	1	36	3	17	56.49	0.76	27.48	2.29	12.98
	Q41.	I replace cannula / catheters for a vessel that fills 72 hours	82	6	30	1	12	62.60	4.58	22.90	0.76	9.16
	Q42.	I do not give under / high-dose medication "abnormal dose"	91	4	24	0	12	69.47	3.05	18.32	0.00	9.16
	Q43.	I do not use the device / tools that I do not know	88	3	23	2	15	67.18	2.29	17.56	1.53	11.45

Regarding the questions that have the highest answer of "Always". It is the most popular answer, but the top questions are: Q1- I put the patient bell in an easy-to-reach place. After that Q15, I check the label information before applying blood or blood products. Also, Q19- I administer the drug once I have verified the patient’s id. Q22: "I record my drug applications. Q23- I prepare drugs according to the aseptic technique for oral drug application. Q29- Before application, I will check if the tool/equipment I will use is working correctly. Last but not least, Q42: "I do not give an "abnormal dose" of medication if it is under- or over-dosed."

Considering the questions with the highest "Never" answer, it is a small number, but it needs to be taken into consideration. A number of these are: Q4- I remove unused materials from the patient’s room, Q5- I lock beds and wheelchairs when not in use, Q11- I monitor the patient after administering antihypertensive and sedative medications, Q17- I prepare blood and blood products according to the procedure, and Q41- I replace cannulas and catheters after 72 hours.

**Fig 1:**
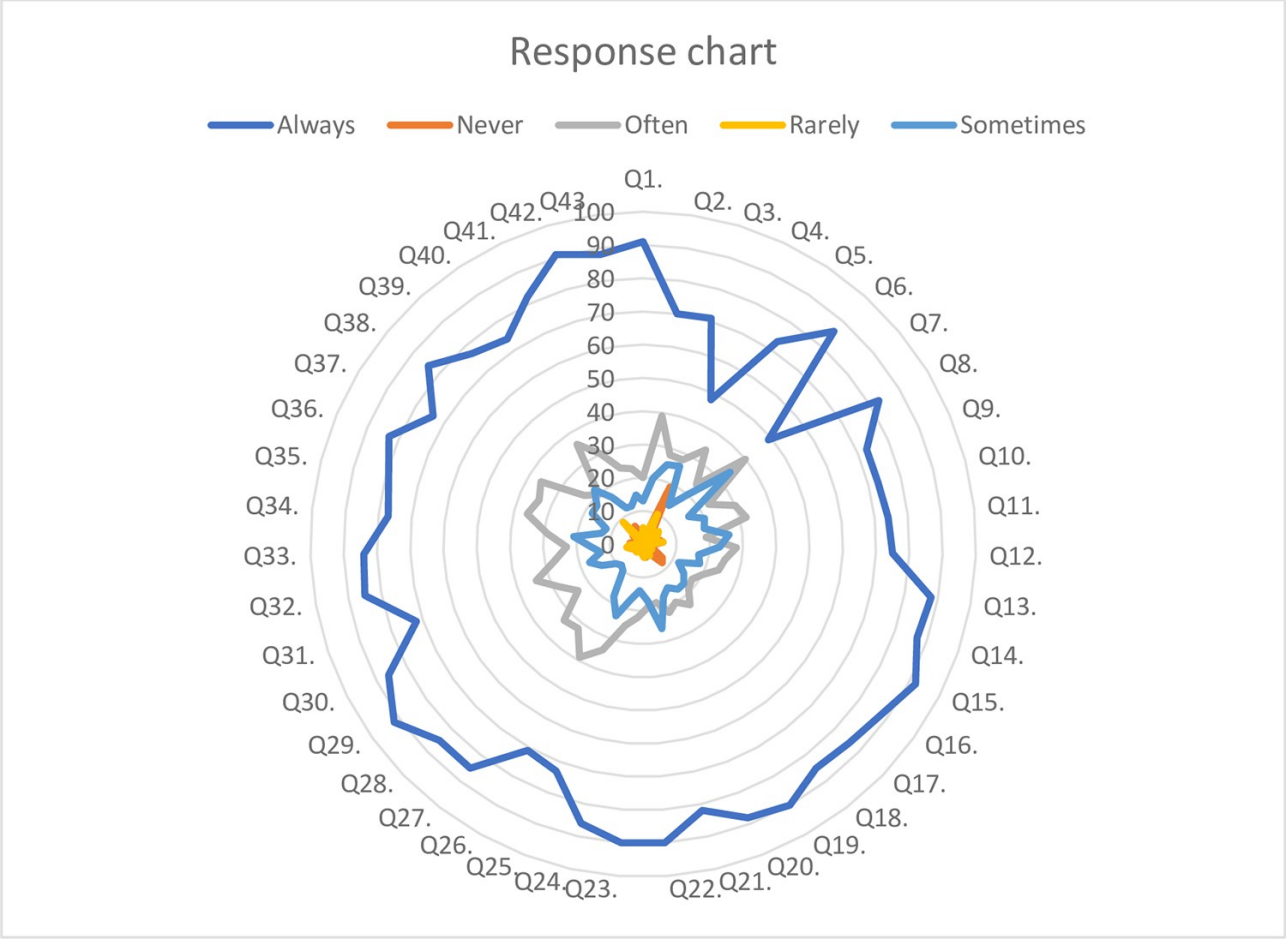
Response types for medical errors practices.

Based on the response chart, it was possible to determine the type of responses received from respondents. The majority of responses are "Always". It is followed by "Often" and "Sometimes". “Never” has the minimum number of responses as shown in Fig 1.

Table 3 represents the frequency and percentage of medical errors made and reported by the interns. It can be seen that medical errors reported and made are same for both “Yes” and “No” category. A total of 105 participants did not make any medical errors and did not report them. Out of 131, 26 participants have made and reported medical errors.

**Table 3:** Medical errors made and reported nursing intern students.

	Frequency		Percentage	
	No	Yes	No	Yes
**Did you make any medical errors?**	105	26	80.15	19.85
**Did you report any medical errors?**	105	26	80.15	19.85

In Table 4, the type of error made by participants is presented. Out of 26, one participant has done mistake by mixing medications, five participants did needle sick injury, three had given wrong dose, fifteen participants wrongly inserted canula, four has done wrong route mistakes.

**Table 4:** Type of errors reported by students.

What is the type of error that you made?	Mix medication	Needle stick injury	None	Wrong Dose	Wrong insertion of cannula	Wrong route
**Frequency**	**1**	**5**	**103**	**3**	**15**	**4**
**Percentage**	0.76	3.82	78.63	2.29	11.45	3.05

In Table 5, the association between the medical errors made by group of participants below and above 4 months experience has been tested by applying chi-square test of association. The obtained gamma value is 0.159. Also, the p-value is 0.631. Similarly, Table 6 shows the association of reporting medical errors between groups having 4-months of experience below and above it. The obtained p-value is 0.2706. It has been found that, there is no significant association between the experience groups of interns’ in making and reporting medical errors for p<0.05 i.e. 5% level of significance.

**Table 5:** Association between experience levels and making medical errors.

Did you make any medical errors?		Yes	No	Association between the experience groups in making medical errors
Experience	Less equal to 4 months	23	89	Gamma = 0.159, Chi-square = 0.23, p-value = 0.631
	Greater than 4 months	3	16	

**Table 6:** Association between experience levels and reporting medical errors.

Did you report any medical errors?		Yes	No	Association between the experience groups in reporting medical errors
Experience	Less equal to 4 months	23	89	Gamma = 0.397, Chi-square = 1.2137, p-value = 0.2706
	Greater than 4 months	3	16	

The Descriptive of the sum of all the responses for each subscale out of **43 items**: 12 items in Factor 1‐Falls, six items in Factor 2‐ Blood and Blood Products Transfusion, six items in Factor 3‐Medication Practices, eight items in Factor 4‐Care Practices, five items in Factor 5‐Communication, and six items in Factor 6‐Other Controlled Practices are TotalF1, TotalF2, TotalF3, TotalF4, TotalF5, and TotalF6, respectively are presented in Table 7. Total score is obtained by the sum of the scores of all the items which is further analyzed for the descriptive. It shows that the mean score is 188. The median and mode are equal and their value is 192. The interquartile range is 30.5, whereas the range is 172. The minimum score obtained is 43 and the maximum is 215.

**Table 7:** The descriptive of total scores.

	TotalF1	TotalF2	TotalF3	TotalF4	TotalF5	TotalF6	Total _ALL
N	131	131	131	131	131	131	131
Mean	50.6	26.6	26.7	35.5	22	26.3	188
Median	51	29	28	37	23	27	192
Mode	50	30	30	40	25	30	192
Standard deviation	7.67	4.65	4.22	5	3.27	4.18	24.9
Range	48	24	24	32	20	24	172
Minimum	12	6	6	8	5	6	43
Maximum	60	30	30	40	25	30	215

Table 8 shows the descriptive for the mean value of scores obtained by dividing the total score of each subscale category by the number of items in the subscale. The mean score lies in between 1 and 5. The highest mean came with F3- Medication Practices. It gives imprison that the students have a lot of information and have many regulations in internship about medication practices. Also, in most of hospitals the quality and continuous education units make regular seminars and workshops about medication practices for new nurses and intern.

**Table 8:** Descriptive statistics for mean scores across subscale categories.

	MeanF1	MeanF2	MeanF3	MeanF4	MeanF5	MeanF6	Mean
N	131	131	131	131	131	131	131
Mean	4.22	4.43	4.45	4.43	4.41	4.39	4.36
Median	4.25	4.83	4.67	4.63	4.6	4.5	4.47
Standard deviation	0.639	0.774	0.704	0.624	0.654	0.696	0.579
Range	4	4	4	4	4	4	4
Minimum	1	1	1	1	1	1	1
Maximum	5	5	5	5	5	5	5

While the lowest mean score of the nursing students on the factor is F1- Falls of the patient. This may interpret as students had less information about falling and its sequences on the patient and it needs to add to the study plan of the nursing department.

The Friedman test result indicates a statistically significant difference between the mean scores of the subscales (χ^2^(5) = 53.1, p < 0.001).

**Fig 2:**
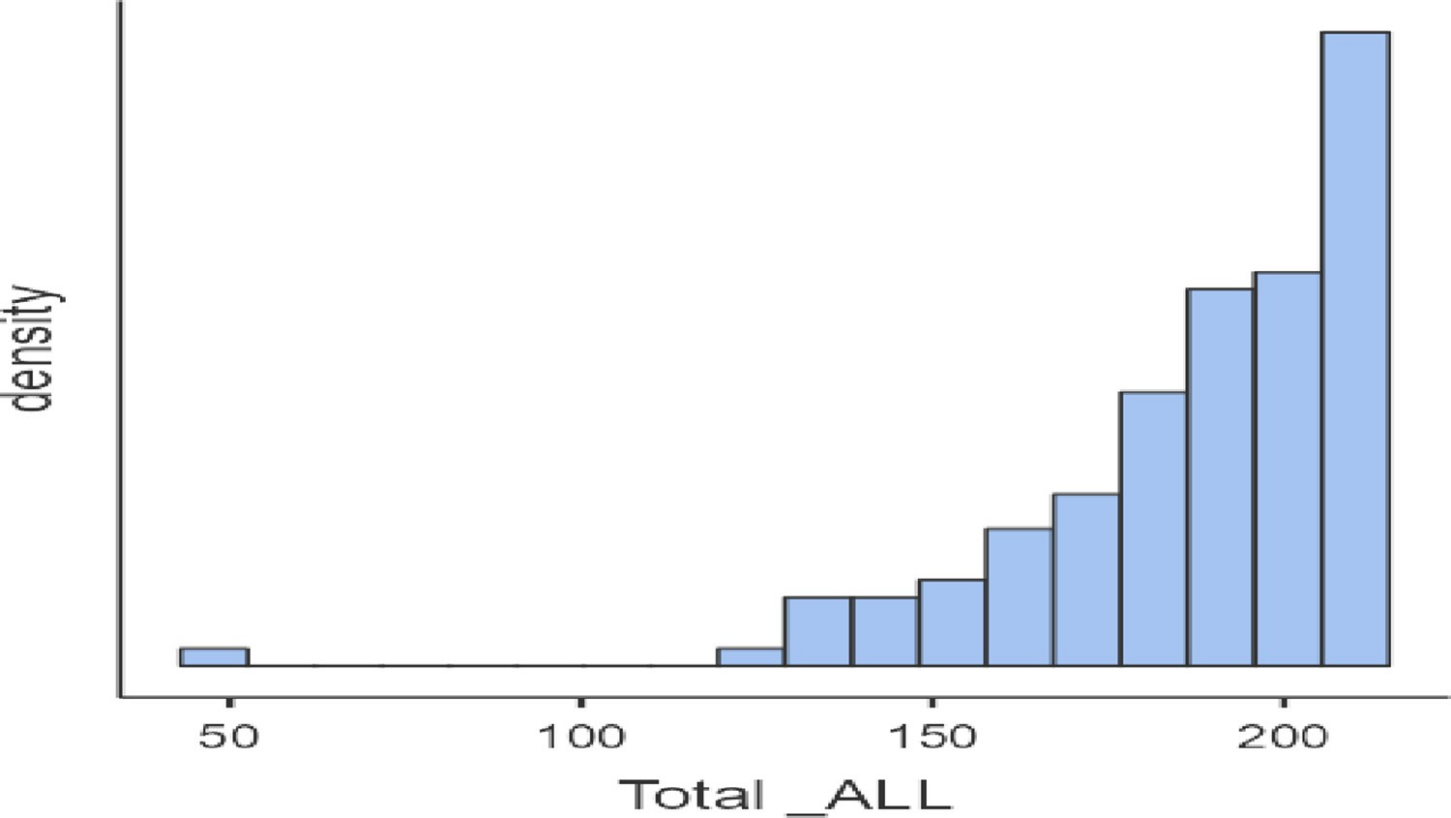
Cautious of students toward medical errors.

To determine whether the intern is cautious of medical errors or not, the range was divided between 43 and 215, i.e. 172 by 2, and considered that above 129 (43+172/2) and below 129 scores show that interns are cautious and not cautious, respectively. It has been found that only one participant with a score of 43 is not cautious, whereas all others come under the cautious intern category. The distribution of density of the Total ALL score obtained is presented in Fig 2.

The types of errors reported by the interns are presented in Table 9. Out of 131, 103 haven’t reported any medical error. Whereas, 28 have reported their medical errors.

**Table 9:** Types of errors reported by nursing intern students.

Types of Error	Frequency	Percent
**None**	103	78.63
**Wrong insertion of the cannula**	15	11.45
**Needle stick injury**	5	3.82
**Wrong Dose**	3	2.29
**Mix medication**	1	0.76
**Wrong route**	4	3.05
**Total**	131	100%

## Discussion

This study aims to evaluate nursing students’ awareness of medical errors during their clinical internship in Al- Ahsa and to identify the types of medical errors that occur during such internship program. Nursing errors are defined as incorrect decisions, omissions, or actions for which nurses are accountable, potentially leading to adverse consequences for the patient [30]. The knowledge and experience acquired during nursing education play a critical role in shaping students’ competence in ensuring and enhancing patient safety in care practices [31].

Nurses are essential members of the healthcare team, playing a pivotal role in patient care due to their responsibilities and being the frontline who interact with the patients and address their various needs [32]. The results revealed that the majority of the students consistently responded with “always” to 43 scale questions with always addressing errors related to falls, blood and blood products transfusion, medication practices, care practices, communication, and other controlled practices. This outcome can be attributed to the fact that most students had sufficient practical experience, with a minimum of one month and a maximum of 12 months of clinical exposure.

In our study, the mean score of nursing students on the whole factor was 4.36, indicating a high level of awareness about medical errors among intern nurses, thus reducing the likelihood of errors. This observation could be attributed to the circumstances under which our study was conducted after the COVID-19 pandemic in Saudi Arabia’s eastern region. Consequently, collecting data from different universities proved to be challenging, which may have imitated the generalizability of our study’s findings. On the whole scale, nursing students scored 188 out of 215. Based on the students’ mean scale sub-dimension scores, it was found that medication practices scored the highest. This can be explained by the fact that our students are exposed to drug calculation in each semester starting from the basic course throughout their education, and this aspect is further emphasized during their practice sessions with their clinical instructors. Similarly, previous studies found that intern nursing students are unlikely to make medical errors [31, 33].

In contrast, Bam (2021) found medication errors to be the most prevalent ME [34]. Medication errors has been associated with inadequate knowledge of procedures and lack of supervision in clinical settings [35]. Furthermore, despite undergoing pharmacology courses as part of their undergraduate education, nursing students’ practical knowledge regarding pharmaceutical therapy application remains is inconsistent and unregulated [36]. Therefore, it is imperative for educational authorities to incorporate techniques into the training program aimed at mitigating potential adverse effects arising from pharmaceutical errors. Nursing students should be thoroughly versed in the importance of administering the right medications [37]. Opportunities for administering medication in a clinical context are not always consistent and may vary depending on the facility and the guidelines of nursing program [38].

Falls of patients is the factor with the lowest score among nursing students. This can be attributed to the fact that nursing students are educated about safety measures, including falls prevention, only in the fundamental course. During their foundational nursing education, nursing students receive instruction on patient safety, which includes fall prevention. However, the clinical training curriculum does not discuss falls or the risk of falls [39].

Our students ranked fall as the least important factor in our study. In contrast to our findings, another study found medication errors to be the most common type of ME [40, 41]. For example, a study conducted by Cooper in the USA found that both nurses and nursing students expressed equal concern about errors, with nursing students more likely to report errors if they posed a potential harm to patients.

Our data do not align with certain findings in the literature, such as Altuntaş et al, (2019) claim that nursing students tend to underestimate medical errors [42]. Moreover, another study found that senior students commit more drug administration errors compared to younger students [43].

According to our findings, lower workloads and supportive work environments permit students to provide nursing care without making medical errors. Additionally, in our study worked exclusively morning shifts under the direct supervision of clinical instructors. The study participants exhibited a low tendency to make medical errors, which is a positive finding when considering strategies for enhancing patient safety procedures. Nursing students may be particularly concerned about errors, and they tend to experience more anxiety when these errors have the potential to cause harm to patients.

Similar to our findings, Musharyanti et al. (2019) [44], suggested that inadequate role models and inadequate supervision contribute to medication errors. Additionally, a lack of knowledge regarding safety and its integration into nursing education was found to be associated with medical errors and compromised patient safety [45].

However, our study results do not in align with those of studies conducted in several other countries. One study suggested that a lack of training on medication safety during the preclinical phase prevented students from fully understanding their role in medication safety. Nursing students may struggle to bridge the gap between theoretical knowledge of patient safety and its practical application in clinical settings [46]. Similarly, an Iranian study found that there was no follow-up patient safety training after the first semester, which hinder the development of necessary safety skills [47].

The current study shows that the majority of students reported no medication errors during their clinical practice. However, most of them did not report errors when they occurred. The most commonly reported error was the incorrect insertion of a cannula, while was the least common was mixing medications. Additionally, there was no significant association between the interns’ experience levels and the likelihood of making or reporting medical errors. These findings align with Alser et al. (2020), who also found no significant association between years of experience or gender and students’ attitudes toward patient safety [20].

The present study aligns with Birgili & Şahin, (2019), who reported that nursing students’ tendencies to make medical errors were low [32]. Abukhalil et al. (2022) supported our findings, revealing differences in healthcare professionals’ awareness of medication errors. The study highlights the importance of implementing appropriate policies to educate healthcare professionals about medication errors. To further enhance drug safety, a national medication error reporting system and regulatory framework should be established [48]. Aljadhey et al. (2016) found that adverse events were common in Saudi hospitals, particularly in ICUs, where they contributed to significant morbidity and mortality [13]. Future research should focus on identifying and evaluating interventions aimed at reducing medication-related harms. Additionally, nursing curriculum should place greater emphasis on increasing students’ awareness and knowledge of adverse drug events during their clinical practice, equipping them to provide safe patient care in their future careers.

Biresaw et al. (2020) reported that factors as age, level of education, working experience, training on patient safety, and information provided during continuing education were significantly associated with nurses’ knowledge regarding patient safety [49]. Hospitals must continue to assess the perceived barriers among nurses using questionnaires such as the MER Barriers and work towards fostering a culture that encourage better reporting. Innovative methods are needed to engage nurses in discussions about the relationship between error reporting, performance improvement and patient outcomes [50]. Safarpour et al (2017) reported that, although nursing students had a positive attitude toward reporting medical errors, their knowledge to medical errors and reporting procedures was limited [51].

Alrabadi et al. (2021) emphasize that nurses are central to the clinical environments, and must work collaboratively as an integrated team to reduce the likelihood of medication errors [52]. To achieve this, it is crucial to systemize protocols, standardizing procedures, adhering to the “five rights” of medication administration, ensuring thorough documentation, maintaining open communication, disclosing medications to patients. Additional measures include improving labeling and packaging format, optimizing the work environment, reducing workload, minimizing distraction, addressing system faults, enhancing job security for nurses, and fostering a blame free culture.

### Limitation and recommendations

Self-administered survey surveys are often considered a limitation due to the potential for biased question interpretation. Additionally, the restriction to data collection from a single region which may have imitated the generalizability of our study’s findings. Nonetheless, identifying strategies and prevention measures for this context can be facilitated by understanding the experiences of students from different settings. Furthermore, there is a need for future research endeavors, such as interventions aimed at enhancing nursing students’ awareness of medical errors or investigating the impact of clinical supervision on error prevention. These initiatives will advance knowledge about practical strategies for enhancing patient safety in nursing practice and education.

### Implications for practice

The findings of this study can be instrumental in developing and implementing educational programs tailored for nursing interns to address medical errors. These programs can improve interns’ understanding of medical errors, their associated risk, and preventative measures. The study’s conclusions underscore the importance of developing and evaluating initiatives aimed at augmenting interns’ awareness of medical errors to improve patient safety. Furthermore, this research holds the potential to inform evidence-based clinical patient safety practices and policy decisions, contingent upon the incorporation of certain methodological and presentation improvements.

## Conclusion

The study found that nursing students demonstrate a low likelihood of committing medical errors. This finding is promising for the development of patient safety procedures. Students may also be concerned about errors, and they are more likely to be fearful when errors can harm patients. Nurses must implement medication safety in clinical settings in order to achieve professional competency in the future. In terms of nursing education, the results of this study can be interpreted positively

### Relevance to clinical practice

It highlights how crucial it is to establish a safety culture early in nursing education. Nurses, as a healthcare professional, play an essential role in the implementing and maintaining medication safety in clinical settings. The positive interpretations of these findings have for nursing education emphasizes the possibility of helping nursing students in developing a safety-oriented mindset. This, in turn, contributes to shaping a future workforce that prioritizes patients’ needs and reduces the likelihood of medical errors.

## References

[1] World Health Organization (a). 10 facts on patient safety. Patient safety fact file. Geneva: WHO; 2019. https://www.who.int/features/factfiles/patient_safety/en/ Accessed 9 Feb 2022

[2] Tarhan M., & Elibol E. (2023). The effect of a brief mindfulness-based stress reduction program on strengthening awareness of medical errors and risks among nursing students. *Nurse Education in Practice*, 70, 103655. doi: 10.1016/j.nepr.2023.103655 37167800

[3] Dehvan F, Nobahar M, Razavi MR, Ghorbani R. Assessment of medication errors and factors affecting its occurrence in intensive critical care units of semnan city hospitals. J Iran Soc Anaesthesiol Intensive Care. 2015;37:172–81.

[4] Madady Z. Nursing medication errors: causes and solutions (a review study). Journal of Hospital. 2015 Sep 10;14(3):101–10.

[5] World Health Organization (b). Patient safety- global action on patient safety. Report by the director-general. Geneva: World Health Organization; 2019. https://apps.who.int/gb/ebwha/pdf_files/WHA72/A72_26-en.pdf, Accessed 10 March 2022

[6] Farzi S, Irajpour A, Saghaei M, Ravaghi H. Causes of Medication Errors in Intensive Care Units from the Perspective of Healthcare Professionals. J Res Pharm Pract. 2017 Jul-Sep;6(3):158–165. doi: 10.4103/jrpp.JRPP_17_47 29026841 PMC5632936

[7] Carver N, Gupta V,Hipskind JE. Medical Error. [Updated 2021 Feb 16]. In: StatPearls [Internet]. Treasure Island (FL): StatPearls Publishing; 2021 Jan-. Available from: https://www.ncbi.nlm.nih.gov/books/NBK430763/

[8] Gold KB. Medical students’ exposure and response to error on the wards.

[9] Mohsin SU, Ibrahim Y, Levine D. Teaching medical students to recognise and report errors. BMJ Open Quality. 2019 Jun 1;8(2):e000558. doi: 10.1136/bmjoq-2018-000558 31276054 PMC6579567

[10] Stratton KM, Blegen MA, Pepper G, Vaughn T. Reporting of medication errors by pediatric nurses. Journal of pediatric nursing. 2004 Dec 1;19(6):385–92. doi: 10.1016/j.pedn.2004.11.007 15637579

[11] Kaushal R, Bates DW, Landrigan C, McKenna KJ, Clapp MD, Federico F, et al. Medication errors and adverse drug events in pediatric inpatients. Jama. 2001 Apr 25;285(16):2114–20. doi: 10.1001/jama.285.16.2114 11311101

[12] Almalki ZS, Alqahtani N, Salway NT, Alharbi MM, Alqahtani A, Alotaibi N, et al. Evaluation of medication error rates in Saudi Arabia: A protocol for systematic review and meta-analysis. Medicine. 2021 Mar 5;100(9):e24956. doi: 10.1097/MD.0000000000024956 33655962 PMC7939210

[13] Aljadhey H, Mahmoud MA, Ahmed Y, Sultana R, Zouein S, Alshanawani S, et al. Incidence of adverse drug events in public and private hospitals in Riyadh, Saudi Arabia: the (ADESA) prospective cohort study. BMJ open. 2016 Jul 1;6(7):e010831. doi: 10.1136/bmjopen-2015-010831 27406640 PMC4947792

[14] Nie Y, Li L, Duan Y, Chen P, Barraclough BH, Zhang M, et al. Patient safety education for undergraduate medical students: a systematic review. BMC medical education. 2011 Dec;11:1–8. 21669007 10.1186/1472-6920-11-33 PMC3128569

[15] Fathi A, Hajizadeh M, Moradi K, Zandian H, Dezhkameh M, Kazemzadeh S, et al. Medication errors among nurses in teaching hospitals in the west of Iran: what we need to know about prevalence, types, and barriers to reporting. Epidemiology and health. 2017;39. doi: 10.4178/epih.e2017022 28774169 PMC5543300

[16] Jember A, Hailu M, Messele A, Demeke T, Hassen M. Proportion of medication error reporting and associated factors among nurses: a cross sectional study. BMC nursing. 2018 Dec;17:1–8. 29563855 10.1186/s12912-018-0280-4 PMC5848571

[17] Dehvan F, Mokhtari Z, Aslani M, Ebtekar F, Gheshlagh RG. The prevalence of needlestick injury in students of medical sciences universities: a systematic review and meta-analysis. Hayat. 2018;24(2).

[18] Crigger NJ, Meek VL. Toward a theory of self‐reconciliation following mistakes in nursing practice. Journal of Nursing scholarship. 2007 Jun;39(2):177–83. doi: 10.1111/j.1547-5069.2007.00164.x 17535319

[19] Robertson JJ, Long B. Suffering in silence: medical error and its impact on health care providers. The Journal of emergency medicine. 2018 Apr 1;54(4):402–9. doi: 10.1016/j.jemermed.2017.12.001 29366616

[20] Alser M, Böttcher B, Alfaqawi M, Jlambo A, Abuzubaida W, Abu-El-Noor N. Undergraduate medical students’ attitudes towards medical errors and patient safety: a multi-center cross-sectional study in the Gaza Strip, Palestine. BMC Medical Education. 2020 Dec;20:1–9. doi: 10.1186/s12909-020-02375-z 33213439 PMC7678054

[21] Weaver SJ, Lubomksi LH, Wilson RF, Pfoh ER, Martinez KA, Dy SM. Promoting a culture of safety as a patient safety strategy: a systematic review. Annals of internal medicine. 2013 Mar 5;158(5_Part_2):369–74. doi: 10.7326/0003-4819-158-5-201303051-00002 23460092 PMC4710092

[22] Ali LA, Saifan AR, Alrimawi I, Atout M, Salameh B. Perceptions of nurses about reporting medication administration errors in Jordanian hospitals: a qualitative study. Applied Nursing Research. 2021 Jun 1;59:151432. doi: 10.1016/j.apnr.2021.151432 33947517

[23] GMC. Education standards, Guidance and Curricula [Internet]. Gmc-uk.org. 2019. Available from: https://www.gmc-uk.org/education/standards-guidance-and-curricula

[24] Vaismoradi M, Salsali M, Marck PJ. Patient safety: nursing students’ perspectives and the role of nursing education to provide safe care. International Nursing Review. 2011 Dec;58(4):434–42. doi: 10.1111/j.1466-7657.2011.00882.x 22092321

[25] Lee SE, Quinn BL. Incorporating medication administration safety in undergraduate nursing education: A literature review. Nurse education today. 2019 Jan 1;72:77–83. doi: 10.1016/j.nedt.2018.11.004 30453203

[26] Oh JW, Kim JE. Effectiveness of a virtual reality application‐based education programme on patient safety management for nursing students: A pre‐test–post‐test study. Nursing Open. 2023 Dec;10(12):7622–30. doi: 10.1002/nop2.2001 37767936 PMC10643842

[27] Salameh BS, Salameh BS. Self-confidence and satisfaction among nursing students with the use of high fidelity simulation at Arab American University, Palestine. International Journal of Health and Life-Sciences. 2017 Sep 1;3(2):15–23.

[28] Sherwood G, Barnsteiner J, editors. Quality and safety in nursing: A competency approach to improving outcomes. John Wiley & Sons; 2021 Dec 13.

[29] Öztürk H, Kahriman I. Development of a medical error scale for nurses in Turkey. Eastern Mediterranean Health Journal. 2020 May 1;26(5). doi: 10.26719/emhj.19.025 32538445

[30] Eltaybani S, Abdelwareth M, Abou‐Zeid NA, Ahmed N. Recommendations to prevent nursing errors: Content analysis of semi‐structured interviews with intensive care unit nurses in a developing country. Journal of nursing management. 2020 Apr;28(3):690–8. doi: 10.1111/jonm.12985 32104934

[31] TÜRK G, ÖZDEMİR S, KOCAÇAL GÜLER E İntörn Hemşirelerin Tıbbi Hata Eğilimlerinin İncelenmesi. Turkiye Klinikleri Journal of Nursing Sciences. 2019 Oct 1;11(4).

[32] FatmaBirgili RN, Şahin M. Determination of nursing students’ medical errors. International Journal of Nursing. 2019 Jun;6(1):25–32.

[33] Gunes U, Zaybak A, Baran L, Ozdemir H. Determining the tendency levels of intern nurses toward medical errors. Ege University J Nursing Faculty. 2016;32:41–9.

[34] Bam V, Safowaa A, Lomotey AY, Nkansah AS. Nursing students’ perception of medical errors: A cross‐sectional study in a university. Nursing Open. 2021 Nov;8(6):3152–60. doi: 10.1002/nop2.1028 34363437 PMC8510776

[35] Mousavi SK, Kamali M. Effect of the peer mentoring method on medication errors in nursing students. Journal of Medical Education Development. 2024 Apr 10;17(53):18–26.

[36] Khalili Z, Molavi Vardanjani M, Shamsizadeh M, Alimohammadi N, Tohidi S, Fallahinia G, et al. Medication errors in nursing students. Scientific Journal of Nursing, Midwifery and Paramedical Faculty. 2018 Jan 10;3(3):8–16.

[37] Dionisi S, Giannetta N, Liquori G, De Leo A, D’Inzeo V, Orsi GB, et al. Medication errors in intensive care units: An umbrella review of control measures. InHealthcare 2022 Jun 29 (Vol. 10, No. 7, p. 1221). MDPI. doi: 10.3390/healthcare10071221 35885748 PMC9320368

[38] Jarvill M, Neubrander J, Kim M. Nursing student medication administration practice in the clinical setting: a descriptive study. Journal of Nursing Education. 2022 Mar 1;61(3):137–42. doi: 10.3928/01484834-20211128-09 35254164

[39] Kim MH, Jeon HW, Chon MY. Study on the knowledge and attitudes of falls and awareness of fall risk factors among nursing students. Indian Journal of Science and Technology. 2015 Jan 1;8:74.

[40] Cooper EE. Nursing students’ perception of safety in clinical settings: From the quality and safety officer.

[41] Muroi M, Shen J, Angosta A. Association of medication errors with drug classifications, clinical units, and consequence of errors: Are they related? Applied Nursing Research. 2017; 33: 180–185. doi: 10.1016/j.apnr.2016.12.002 28096015

[42] Altuntaş S, Güven G, Öztürk K, Işık E. HEMŞİRELİK ÖĞRENCİLERİNİN TIBBİ HATALARA KARŞI TUTUMLARI. Bandırma Onyedi Eylül Üniversitesi Sağlık Bilimleri ve Araştırmaları Dergisi. 2019 Jun 6;1(1):1–9.

[43] Zaybak A, Taşkıran N, Telli S, Ergin EY, Şahin M. The opinions ofnursing students regarding sufficiency of their drug administrationknowledge. Journal of Education and Research in Nursing 2017; 14(1): 6–13.

[44] Musharyanti L, Claramita M, Haryanti F, Dwiprahasto I. Why do nursing students make medication errors? A qualitative study in Indonesia. Journal of Taibah University Medical Sciences. 2019 Jun 1;14(3):282–8. doi: 10.1016/j.jtumed.2019.04.002 31435418 PMC6694917

[45] Christiansen A, Prescott T, Ball J. Learning in action: developing safety improvement capabilities through action learning. Nurse Education Today. 2014 Feb 1;34(2):243–7. doi: 10.1016/j.nedt.2013.07.008 23938091

[46] Adhikari R, Tocher J, Smith P, Corcoran J, MacArthur J. A multi-disciplinary approach to medication safety and the implication for nursing education and practice. Nurse education today. 2014 Feb 1;34(2):185–90. doi: 10.1016/j.nedt.2013.10.008 24219921

[47] Sulosaari V, Kajander S, Hupli M, Huupponen R, Leino-Kilpi H. Nurse students’ medication competence—an integrative review of the associated factors. Nurse Education Today. 2012 May 1;32(4):399–405. doi: 10.1016/j.nedt.2011.05.016 21652125

[48] Abukhalil AD, Amer NM, Musallam LY. Medication error awareness among health care providers in Palestine: A questionnaire-based cross-sectional observational study. Saudi Pharmaceutical Journal. 2022 Apr 1;30(4):470–7. doi: 10.1016/j.jsps.2022.01.014 35527828 PMC9068552

[49] Biresaw H, Asfaw N, Zewdu F. Knowledge and attitude of nurses towards patient safety and its associated factors. International Journal of Africa Nursing Sciences. 2020 Jan 1;13:100229.

[50] Rutledge DN, Retrosi T, Ostrowski G. Barriers to medication error reporting among hospital nurses. Journal of clinical nursing. 2018 May;27(9–10):1941–9. doi: 10.1111/jocn.14335 29495119

[51] Safarpour H, Tofighi M, Malekyan L, Bazyar J, Varasteh S, Anvary R. Patient safety attitudes, skills, knowledge and barriers related to reporting medical errors by nursing students. International Journal of Clinical Medicine. 2017 Jan 20;8(1):1–1.

[52] Alrabadi N, Shawagfeh S, Haddad R, Mukattash T, Abuhammad S, Al-rabadi D, et al. Medication errors: a focus on nursing practice. Journal of Pharmaceutical Health Services Research. 2021 Mar 1;12(1):78–86.

